# Effects of Differentiation *Plasmodiophora brassicae* Physiological Races on the Rhizosphere Microbial Community Structure of Oilseed Rape

**DOI:** 10.3390/microorganisms14040742

**Published:** 2026-03-26

**Authors:** Zijin Hu, Zhongmei Zhang, Xiaoqin Huang, Yaoying Yu, Yue Deng, Pei Song, Yong Liu, Lei Zhang, Xiaoxiang Yang

**Affiliations:** 1 Institute of Plant Protection, Sichuan Academy of Agricultural Sciences, Chengdu 610066, China; huzijinwh@163.com (Z.H.);; 2 Key Laboratory of Integrated Pest Management on Crops in Southwest, Ministry of Agriculture and Rural Affairs, Chengdu 610066, China

**Keywords:** nitrogen cycle, *Brassica napus* L., clubroot, rhizosphere microbiome

## Abstract

Clubroot caused by *Plasmodiophora brassicae* is a devastating soil-borne disease of oilseed rape, and physiological race differentiation of the pathogen greatly hinders disease control. The differential regulatory mechanisms of different *P. brassicae* races on the rhizosphere microecology remain unclear. This study aimed to reveal the race-specific effects of *P. brassicae* on the rhizosphere microenvironment, microbial community and nitrogen cycling of oilseed rape. A pot inoculation experiment was conducted with two typical races from Sichuan Province (race 4 CZ and race 2 KD), combined with soil physicochemical determination, high-throughput sequencing and functional prediction. The results showed that CZ exhibited a higher infection rate but a lower disease index than KD. Both races significantly decreased soil pH and reshaped soil nutrient profiles. Notably, CZ treatment caused a more pronounced pH decrease and was characterized by NH_4_^+^-N accumulation, whereas KD treatment was dominated by NO_3_^−^-N enrichment. Bacterial alpha diversity was increased by both races, following the order KD > CZ > CK. In contrast, fungal alpha diversity was decreased by both races, showing the pattern CK > KD > CZ. Distinct rhizosphere microbial community structures were formed under different race infections, and both races reduced the abundance of nitrogen-fixing bacteria and related functional genes. These findings indicate that distinct *P. brassicae* races shape race-specific rhizosphere microenvironments by differentially regulating soil acidification, nutrient availability and nitrogen-cycling functional microorganisms, thereby driving divergent pathogenic outcomes. This study is the first to reveal differential regulation of the rhizosphere microecology by distinct physiological races of *P. brassicae*, offering new insights for region-specific management of clubroot disease.

## 1. Introduction

Oilseed rape (*Brassica napus* L.) is the largest oilseed crop in China, with an annual planting area exceeding 6.67 million hectares and a seed yield of approximately 4.5 million tons [1]. Clubroot, a soil-borne disease caused by *Plasmodiophora brassicae*, is one of the most devastating diseases affecting oilseed rape production. The resting spores of this pathogen can survive in soil for more than 10 years. After infection, *P. brassicae* induces tumor formation in the root system, seriously impairing water and nutrient uptake. Yield losses in severely diseased fields can exceed 60% and may even result in total harvest failure [2,3,4]. In recent years, driven by climate change and changes in cultivation methods (e.g., no-tillage cultivation and straw return), the geographic distribution and severity of clubroot have expanded, making it a major constraint on the sustainable development of China’s oilseed rape industry.

*P. brassicae* exhibits pronounced physiological race differentiation [5,6]. Research based on the Williams differential system has identified races 2, 4, 7, and 11 in China, with race 4 being the dominant group. These races differ significantly in pathogenicity and environmental adaptability [7,8]. However, the mechanisms underlying these differences remain poorly understood. The rhizosphere microdomain, as the core interface of plant-microbe–soil interactions, regulates disease occurrence through various pathways, including nutrient cycling by microbial communities, secretion of antagonistic compounds, and induction of plant systemic resistance [9,10]. Therefore, systematically investigating how different physiological races of *P. brassicae* regulate the rhizosphere microbial community is critical for elucidating race-specific pathogenicity and regional prevalence, and for developing targeted ecological control strategies.

Considerable progress has been made in understanding interactions between *P. brassicae* and rhizosphere microorganisms. Using 16S rRNA sequencing, Xi et al. showed that *P. brassicae* inoculation significantly increased bacterial diversity in the rhizosphere of susceptible Chinese cabbage, increased the relative abundance of Proteobacteria, and decreased that of Acidobacteria, indicating a close association between bacterial community structure and clubroot development [11]. Kang et al. further demonstrated that *P. brassicae* infection reduces soil pH and increases organic matter content; these physicochemical changes promote the enrichment of fungal communities (e.g., *Fusarium*), thereby exacerbating disease severity [12]. Nitrogen, a central element in soil ecosystems, plays a critical role in regulating plant–pathogen–soil microbe interactions [13]. Previous study indicated that several nitrogen cycle-associated bacterial genera, including *Nitrosomonas*, *Tumebacillus*, and *Halomonas*, were significantly downregulated in clubroot-susceptible cultivars infected by *P. brassicae*, but upregulated in resistant cultivars. Correspondingly, nitrogen cycle functional analysis revealed that the nitrification-related gene *nxrB* was upregulated in infected susceptible cultivars, whereas genes involved in assimilatory nitrate reduction (*nasA*, *narB*, and *nirA*) were upregulated in infected resistant cultivars [14]. However, most existing studies have not addressed the specificity of interactions between different physiological races and the rhizosphere microenvironment, and systematic comparative analyses are still lacking.

Given the substantial differences in pathogenic mechanisms and ecological adaptability among physiological races of *P. brassicae*, and the limited understanding of their race-specific interactions with rhizosphere microorganisms, a systematic study was conducted to clarify their differential regulatory effects on the oilseed rape rhizosphere microbial community. Specifically, this study focused on the following questions: (1) Do different physiological races differentially affect rhizosphere soil acidification and nutrient forms? (2) Do they exert distinct effects on the diversity of rhizosphere microbial communities? And (3) How do different races reshape microbial community structure, and which key taxa (e.g., Proteobacteria and Ascomycota) respond most strongly? These findings are expected to reveal race-specific pathogenic mechanisms from a microecological perspective and provide a theoretical basis for differentiated ecological control of clubroot.

## 2. Materials and Methods

### 2.1. Plant Material and Source of P. brassicae

The plant material used in this study was the *B. napus* cultivar ‘Chuanyou 36’, which is highly susceptible to clubroot and therefore suitable for studying the pathogenic effects of *P. brassicae*. Two physiological races of *P. brassicae*, namely CZ and KD, were isolated from Chengdu (30.43° N, 103.29° E) and Kangding (30.29° N, 101.46° E) in Sichuan Province, respectively, and their identities were confirmed as race 4 and race 2 via the Williams differential host identification system [8]. Resting spores were extracted from diseased root tissues following the method of Li et al. and stored at −20 °C until use [15].

### 2.2. Experimental Design

A greenhouse pot experiment was conducted using healthy garden soil that had never been planted with cruciferous crops. The temperature was set at 22 ± 2 °C with a 16 h light/8 h dark photoperiod and a relative humidity of 60–70%. Plastic pots (15 cm in diameter and 12 cm in height) were filled with 1.5 kg of soil per pot. The soil was inoculated with *P. brassicae* race 4 (CZ) or race 2 (KD) and thoroughly mixed to achieve a final resting spore concentration of 10^6^ spores g^−1^ soil; soil treated with sterile water served as the control (CK). Each treatment consisted of 15 pots. Oilseed rape seeds were surface-sterilized (75% ethanol treatment for 30 s, 2% sodium hypochlorite treatment for 3 min, and rinsed five times with sterile water). The seeds were germinated on sterile, moist filter paper, and uniformly germinated seeds were selected and sown at a density of 10 seeds per pot. When seedlings reached the 2–3 true-leaf stage, they were thinned to five plants per pot.

### 2.3. Disease Survey

Sixty days after oilseed rape planting, when clubroot symptoms were fully expressed, disease incidence was assessed [16]. Disease incidence and disease index were calculated according to the following formulas:

Incidence rate (%) = Number of diseased plants/Total number of investigated plants × 100;

Disease index = (Σ (Number of diseased plants at each stage × Relative value))/(Total number of plants under investigation × Highest incidence of disease) × 100.

### 2.4. Soil Sample Collection

For rhizosphere sampling, plants were carefully removed from the pots, and loosely attached soil was gently shaken off. Soil tightly adhering to the root surface (1–2 mm) was collected using a sterile brush and defined as rhizosphere soil. Rhizosphere soil from five pots within the same treatment was thoroughly mixed to create a composite sample, with three independent replicates per treatment. Approximately 5 g of each composite sample was transferred to a sterile centrifuge tube and stored at −80 °C for subsequent high-throughput sequencing analysis of bacterial and fungal communities.

### 2.5. Analysis of Soil Physicochemical Properties

The soil pH was determined by potentiometry using a FE28 pH meter (Mettler Toledo, Greifensee, Switzerland) at a soil-to-water ratio of 1:2.5 (*w*/*v*), soil organic carbon (SOC) by potassium dichromate oxidation (extractant: 0.8 mol L^−1^ K_2_Cr_2_O_7_-H_2_SO_4_) at 600 nm using a UV-1800 spectrophotometer (Shimadzu, Kyoto, Japan), total nitrogen (TN) by the Kjeldahl method using a Kjeltec 8400 analyzer (Foss, Hillerød, Denmark), alkali-hydrolyzable nitrogen (AN) by alkaline hydrolysis diffusion (soil-to-solution ratio 1:10, extractant: 1 mol L^−1^ NaOH) at 625 nm, total phosphorus (TP) and available phosphorus (AP) by molybdenum-antimony anti-spectrophotometry with HClO_4_-H_2_SO_4_ digestion and 0.5 mol L^−1^ NaHCO_3_ (pH 8.5) extraction respectively (both at 700 nm, same spectrophotometer as SOC), total potassium (TK) and available potassium (AK) by flame photometry using an FP641 photometer (Shanghai Precision Instrument Co., Ltd., Shanghai, China) with HNO_3_-HClO_4_ digestion and 1 mol L^−1^ NH_4_OAc (pH 7.0) extraction respectively, nitrate nitrogen (NO_3_^−^-N) by ultraviolet spectrophotometry (extractant: 0.01 mol L^−1^ CaCl_2_) at 220/275 nm and ammonium nitrogen (NH_4_^+^-N) by indophenol blue colorimetry (extractant: 2 mol L^−1^ KCl) at 625 nm (both with the same spectrophotometer as SOC), and exchangeable calcium (Ex-Ca) and exchangeable magnesium (Ex-Mg) by atomic absorption spectrophotometry using an AA-6300 instrument (Shimadzu, Japan) with 1 mol L^−1^ NH_4_OAc (pH 7.0) extraction; each measurement was performed in triplicate, all results were reported based on soil dry weight (105 °C, 6 h), quality control included reagent blanks, parallel replicates and standard reference materials (relative deviation ≤ 5%), all analyses were conducted by Chengdu Baihui Biotechnology Co., Ltd., (Chengdu, China).

### 2.6. DNA Extraction, PCR Amplification, and Sequencing

Total DNA was extracted from 0.5 g rhizosphere soil using the E.Z.N.A.^TM^ Mag-Bind Soil DNA Kit (Omega Bio-tek, Norcross, GA, USA). Three biological replicates were set for each treatment (CK, CZ, KD), with a total of 9 samples for sequencing. DNA integrity was initially assessed by 1% agarose gel electrophoresis, and concentration and purity were determined using a NanoDrop spectrophotometer (A260/A280 ≥ 1.8). The purity and concentration of extracted DNA were detected by 1% agarose gel electrophoresis and a NanoDrop 2000 spectrophotometer (Thermo Scientific, Waltham, MA, USA), respectively. The V3~V4 region of the bacterial 16S rRNA gene was amplified using primers 341F/806R [17], and the fungal ITS1 region was amplified using primers ITS1F/ITS2R [18]. PCR reactions were carried out with 15 µL of Phusion^®^ High-Fidelity PCR Master Mix (New England Biolabs, Ipswich, MA, USA); 2 µM of forward and reverse primers, and about 10 ng template DNA. Thermal cycling consisted of initial denaturation at 98 °C for 1 min, followed by 30 cycles of denaturation at 98 °C for 10 s, annealing at 50 °C for 30 s, and elongation at 72 °C for 30 s. Finally 72 °C for 5 min. PCR amplicons were detected by 2% agarose gel electrophoresis, and the target bands were recovered and purified using the Qiagen Gel Extraction Kit (Qiagen, Hilden, Germany).

Sequencing libraries were generated using TruSeq^®^ DNA PCR-Free Sample Preparation Kit (Illumina, San Diego, CA, USA) following the manufacturer’s recommendations and index codes were added. The library quality was assessed on the Qubit@ 2.0 Fluorometer (Thermo Scientific, Waltham, MA, USA) and Agilent Bioanalyzer 2100 system. Finally, the library was sequenced on an Illumina NovaSeq platform and 250 bp paired-end reads were generated.

### 2.7. Statistical Methods

For the pathogenicity experiment indicators (disease incidence and disease index), the disease incidence data were arcsine square root-transformed, and all data were analyzed by one-way ANOVA followed by Duncan’s new multiple range test (agricolae package) at *p* < 0.05 using R software (v4.2.1).

Raw sequencing data were quality-filtered using QIIME (V1.9.1) [19]. Clean reads were clustered into Operational Taxonomic Units (OTUs) at 97% similarity using Uparse software (v7.0.1001) [20]. Species annotation was performed using the Silva Database [21]. Alpha diversity indices, including ACE, Chao1, Shannon, Simpson, and PD whole tree, as well as beta diversity using PCoA based on the Bray–Curtis distance, were calculated using QIIME2 (v2022.8) [22]. Inter-group differences were analyzed using one-way ANOVA followed by Tukey’s HSD test (*p* < 0.05). Functional prediction of nitrogen-cycling genes was performed using PICRUSt2 (v2.3.0) [23].

The raw sequence data reported in this paper have been deposited in the Genome Sequence Archive [24] in the National Genomics Data Center [25], China National Center for Bioinformation/Beijing Institute of Genomics, Chinese Academy of Sciences (GSA: CRA037306), which are publicly accessible at https://ngdc.cncb.ac.cn/gsa (accessed on 19 January 2026).

## 3. Results

### 3.1. Effects of Different P. brassicae Physiological Races on Clubroot Incidence

**Figure 1:**
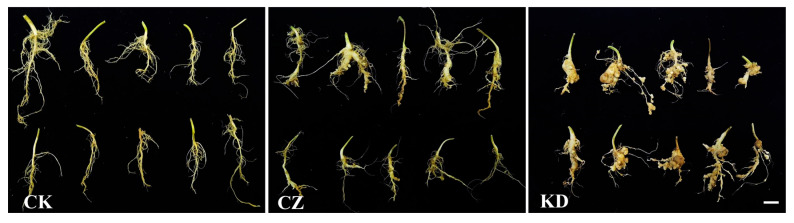
Root galls of clubroot disease induced by inoculation with different physiological races of *P. brassicae*. CK, CZ and KD represent the control group, *P. brassicae* race 4 (CZ) treatment group and race 2 (KD) treatment group, respectively. The same below. Scale bar = 2 cm.

**Figure 2:**
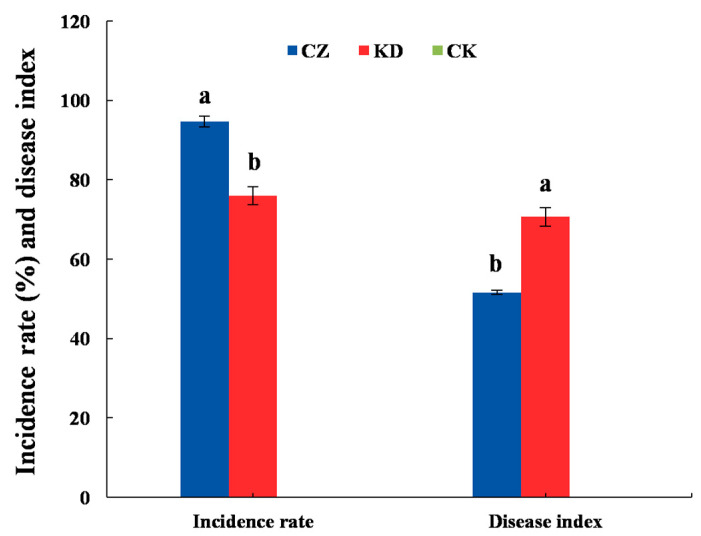
Disease incidence and disease index of clubroot in oilseed rape. Different lowercase letters above the columns indicate significant differences among treatments (*p* < 0.05).

The control group (CK) exhibited 0% disease incidence and no visible clubroot symptoms throughout the experiment. Inoculation with *P. brassicae* race 4 (CZ) and race 2 (KD) both successfully induced typical clubroot formation in oilseed rape (Figure 1 and Figure 2). The incidence rate of race 4 (CZ) reached 94.67%, which was significantly higher than the 76.00% observed for race 2 (KD) (*p* < 0.05). In contrast, the disease index of KD (70.67) was significantly higher than that of CZ (51.62) (*p* < 0.05) (Figure 2). These results indicate that race 4 (CZ) exhibits a relative “high infection rate—low disease index” pattern, while race 2 (KD) shows a “low infection rate—high disease index” pattern.

### 3.2. Effects of Different Races Treatment on Soil Physicochemical Properties

Inoculation with *P. brassicae* significantly altered the physicochemical environment of the oilseed rape rhizosphere soil (Table 1). Compared with the CK group, both CZ and KD treatments caused significant soil acidification (*p* < 0.05), with the CZ treatment producing the lowest pH. Overall, nutrient contents in the rhizosphere increased in the inoculated groups, but the enrichment patterns differed between races. The CZ treatment had the highest TK and NH_4_^+^-N content, whereas the KD treatment showed significantly higher TN, TP, NO_3_^−^-N, Ex-Ca, and Ex-Mg content compared with other treatments. These results suggested that different physiological races of *P. brassicae* differentially regulate rhizosphere acidification and nutrient turnover, which may further affect microbial community structure and function.

### 3.3. Richness and Diversity of Rhizosphere Bacteria and Fungi in Oilseed Rape Inoculated with Different P. brassicae Races

**Figure 3:**
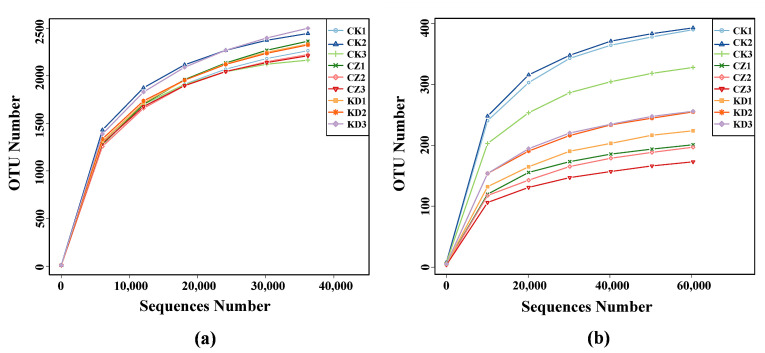
Rarefaction curves of bacteria (**a**) and fungi (**b**) in rhizosphere soil. The x-axis represents the number of sequencing reads, and the y-axis represents the number of observed operational taxonomic units (OTUs); CK1/2/3, CZ1/2/3 and KD1/2/3 represent the three biological replicates of the control group, *P. brassicae* race 4 (CZ) treatment group and race 2 (KD) treatment group, respectively.

Rarefaction curve analysis (Figure 3) showed that with increasing sequence number, the curves for both bacteria and fungi reached a plateau, with sequencing coverage ranging from 98.83% to 99.99% (Table 2). This indicated that the sequencing depth was sufficient to capture the overall composition of the rhizosphere microbial community.

Analysis of bacterial diversity revealed that infection with different physiological races of *P. brassicae* significantly affected the richness and diversity of the rhizosphere bacterial community (Table 2). Compared with the CK group, the ACE and Chao1 indices, which reflect species richness, were obviously increased in the inoculated groups (CZ and KD). Moreover, all measured indices (ACE, Chao1, Shannon, and PD whole tree) were significantly higher in KD than in CZ. In contrast, fungal community diversity showed an opposite pattern. The ACE, Chao1, Shannon, Simpson, and PD whole tree indices of rhizosphere fungi were significantly lower in the inoculated groups (CZ and KD) compared with CK. Among the inoculated treatments, all fungal diversity indices were higher in KD than in CZ.

### 3.4. Rhizosphere Soil Microbial Community Composition

**Figure 4:**
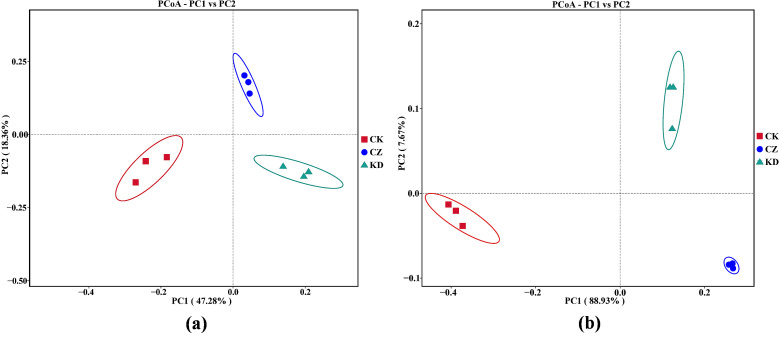
PCoA analysis of bacterial (**a**) and fungi (**b**) communities in rhizosphere soil. Principal Coordinate Analysis (PCoA) of bacterial (**a**) and fungal (**b**) communities in rhizosphere soil based on Bray–Curtis distance. The x-axis and y-axis represent the first and second principal coordinates (PC1 and PC2), with the explained variance of each principal coordinate shown in parentheses.

Principal Coordinate Analysis (PCoA) based on the Bray–Curtis distance showed that the first two principal coordinates (PC1 and PC2) explained 47.28% and 18.36% of the total variance for the bacterial community, and 88.93% and 7.67% of the total variance for the fungal community (Figure 4). The PCoA ordination revealed a clear separation of the bacterial and fungal community structures among the CK, CZ and KD treatment groups in the ordination space. This indicated that infection with different *P. brassicae* physiological races imposed specific selection pressures on the rhizosphere microbial community, resulting in significant restructuring of the microbial community composition.

**Figure 5:**
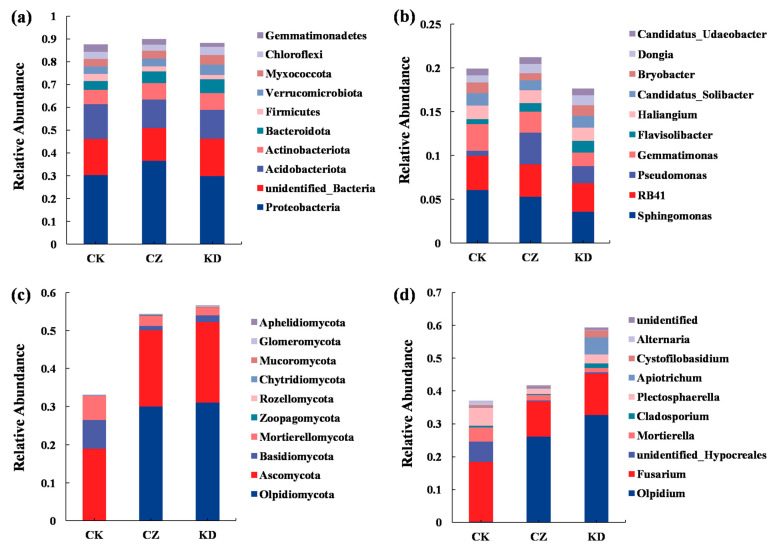
Composition of rhizosphere soil microbial communities. (**a**) Bacterial composition (Phylum level); (**b**) bacterial composition (Genus level); (**c**) fungal composition (Phylum level); (**d**) fungal composition (Genus level).

The dominant bacterial phyla across treatment groups were Proteobacteria, Acidobacteria, Actinobacteria, Bacteroidetes, and unclassified bacteria, collectively accounting for more than 70% of the community (Figure 5a). At the genus level, *Sphingomonas*, RB41, *Pseudomonas*, *Gemmatimonas*, *Flavisolibacter*, and *Haliangium* represented over 70% of identified genera (Figure 5b). Among the different treatments, the relative abundance of Proteobacteria increased markedly in the CZ group, with *Pseudomonas* showing a particularly prominent increase.

The dominant fungal phyla included Olpidiomycota, Ascomycota, Basidiomycota, and Mortierellomycota, together accounting for over 60% of the total community (Figure 5c). At the genus level, the dominant taxa were *Olpidium*, *Fusarium*, unclassified Hypocreales, *Mortierella*, *Cladosporium*, and *Plectosphaerella* (Figure 5d). Infection with *P. brassicae* caused significant shifts: Olpidiomycota increased significantly in the CZ and KD groups, whereas Basidiomycota and Mortierellomycota decreased. At the genus level, *Olpidium* was significantly increased in both inoculated groups. *Cladosporium*, *Apiotrichum*, and *Cystofilobasidium* were most abundant in the KD group, representing the main fungal signatures associated with this race.

### 3.5. Effects on Rhizosphere Soil Nitrogen Cycling

**Figure 6:**
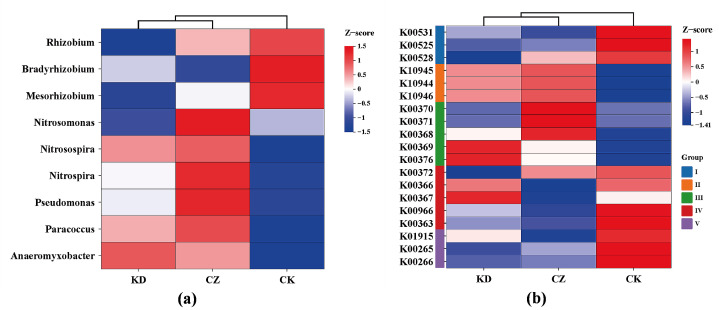
Impact of different *P. brassicae* physiological races on rhizosphere nitrogen cycling. (**a**) Relative abundance of key nitrogen-cycling microbial genera (Z-score normalized); (**b**) Relative abundance of key nitrogen-cycling functional genes (Z-score normalized, I: N-fixation, II: Nitrification, III: Denitrification, IV: Nitrate reduction to ammonium, V: NH_4_^+^ assimilation).

Based on the results above, *P. brassicae* infection significantly altered microbial community structure and nitrogen cycling. The abundance of key nitrogen-cycling microbial genera and functional genes was analyzed. Compared with CK, the relative abundances of nitrogen-fixing *Rhizobium*, *Bradyrhizobium*, and *Mesorhizobium* were significantly decreased in the inoculated treatments. In contrast, the relative abundances of *Nitrosomonas*, *Nitrosospira*, and *Nitrospira* (nitrification-related genera), as well as *Pseudomonas* and *Paracoccus* (denitrification-related genera), followed the pattern CZ > KD > CK (Figure 6a). Functional prediction based on PICRUSt further showed that genes associated with nitrogen fixation, nitrate reduction to ammonium, and ammonia assimilation decreased after inoculation, whereas genes involved in nitrification and denitrification were enriched, also showing the pattern CZ > KD > CK (Figure 6b). These results are consistent with observed differences in soil nitrogen forms (ammonium enrichment in CZ, nitrate enrichment in KD), indicating that *P. brassicae* infection reshaped rhizosphere nitrogen cycling in a race-dependent manner.

## 4. Discussion

This study systematically compared the effects of infection by *P. brassicae* physiological race 4 (CZ) and race 2 (KD) on the rhizosphere microenvironment of oilseed rape using pot experiments combined with high-throughput sequencing. The results demonstrated that different races differ markedly in pathogenicity and in their regulation of soil physicochemical properties and microbial communities. These findings provide a novel microecological perspective for understanding the ecological adaptability and pathogenic mechanisms of different *P. brassicae* races.

### 4.1. Pathogenic Strategies and Ecological Adaptation

The results identified two contrasting pathogenic strategies: race 4 (CZ) displayed a relative “high infection rate—low disease index” pattern, while race 2 (KD) exhibited a “low infection rate—high disease index” lifestyle, which reflects distinct evolutionary and ecological adaptations to their native habitats. From an evolutionary perspective, the Chengdu Plain, the source of CZ, is characterized by intensive farming and continuous cropping. Under such conditions, a “high infection, moderate pathogenicity” strategy may favor long-term persistence and widespread colonization without rapidly killing the host, resembling a form of “gentle parasitism” [26,27]. In contrast, the western Sichuan Plateau, the source of KD, has a short growing season, low temperatures, and strong UV radiation. A “strong pathogenicity” strategy may enable rapid resource acquisition and completion of the life cycle within a limited time window, consistent with adaptation to short-season crops [28,29]. This study provided an ecological and pathological basis for understanding *P. brassicae* distribution and evolution.

### 4.2. Differential Regulation of Rhizosphere Soil Acidification and Nutrient Cycling

Consistent with previous studies, the results confirm that *P. brassicae* infection universally induces rhizosphere soil acidification [12,30]. This acidification likely stems from infection-induced secretion of H^+^ and organic acids [31], and the acidic microenvironment further promotes the resting spore of *P. brassicae* germination [32], forming a positive feedback loop that facilitates disease development. Simultaneously, soil nutrients (N, P, and K) generally increased, likely due to impaired root absorption and rhizosphere accumulation, similar to the effects observed in root-knot nematodes [33].

Notably, the two races differed in N accumulation: the CZ treatment was dominated by NH_4_^+^-N, whereas the KD treatment showed significantly increased NO_3_^−^-N and TN. Consistent with these patterns, N-fixing bacteria (*Rhizobium*) decreased after inoculation, while nitrifying and denitrifying taxa (e.g., *Nitrosospira* and *Pseudomonas*) followed the trend CZ > KD > CK. These microbial shifts mirror the observed changes in soil N forms, suggesting infection shifts nitrogen metabolism toward nitrification/denitrification. Interestingly, although the CK group had the highest predicted abundance of genes associated with N fixation/assimilation, it had the lowest soil N content. This reveals the essence of a healthy rhizosphere: a functional “Microbial Carbon Pump” allowing the plant-microbe system to incorporate N into biomass rather than accumulating it in the soil [34]. In contrast, *P. brassicae* infection—particularly by KD—disrupts host nutrient uptake and microbial nitrogen fixation, leading to ineffective nitrogen accumulation in soil, which is a hallmark of impaired rhizosphere nutrient cycling.

### 4.3. Specific Responses of Microbial Community Structure

*P. brassicae* infection imposed strong selective pressure on the rhizosphere microbial community, leading to significant restructuring of community composition and diversity. Bacterial alpha diversity was significantly enhanced by pathogen inoculation, with the KD treatment showing the highest diversity, which aligns with previous reports that clubroot infection increases rhizosphere bacterial diversity in susceptible hosts [11]. In contrast, fungal alpha diversity was markedly reduced in both inoculated groups, likely due to competitive exclusion driven by the proliferation of the dominant pathogen-associated taxon (Olpidiomycota) (Figure 5c).

PCoA revealed clear separation of microbial communities among CK, CZ, and KD treatments, confirming race-specific effects on community assembly. The CZ treatment was marked by the enrichment of Proteobacteria, particularly the genus *Pseudomonas*. Numerous *Pseudomonas* strains exhibit antagonistic activity or participate in nitrogen metabolism, thereby potentially modulating disease progression [10]. The KD treatment showed increased abundance of *Cladosporium* (a potential pathogenic fungus) [35] and *Cystofilobasidium* (a saprophytic genus) [36], consistent with findings that severely diseased soils tend to accumulate pathogens/saprophytes to aid root “tumor” disintegration and spore dispersal [3].

## 5. Conclusions

In summary, this study is the first to systematically reveal that distinct physiological races of *P. brassicae* drive divergent pathogenic outcomes by differentially reshaping the rhizosphere microenvironment, microbial community structure, and nitrogen-cycling functions of oilseed rape. These findings provide a novel microecological perspective for clarifying the pathogenic differentiation and ecological adaptation of the clubroot pathogen and establish a robust theoretical basis for the region-specific green integrated management of oilseed rape clubroot.

Nevertheless, this study has several limitations. First, the pot experiments were performed under controlled greenhouse conditions with only one soil type and one oilseed rape cultivar, which restricted the generalizability of the findings; thus, multi-soil type, multi-variety validation and multi-site field trials are required to confirm the consistency and repeatability of the observed race-specific regulatory effects in natural environments. Second, only two physiological races were investigated, and the microbial functions were predicted bioinformatically rather than directly detected. Third, the physiological parameters of above-ground plant parts (e.g., chlorophyll a/b content, photosynthetic characteristics, plant morphometry) and soil oxygen content were not determined in this study, which limits the comprehensive understanding of the holistic response of oilseed rape to infection by different *P. brassicae* races.

Therefore, future research should expand the types of *P. brassicae* races, conduct experiments with different soil types and cultivars, carry out multi-site field trials, and combine metagenomics, metabolomics and other methods to directly identify and verify the related microbial functions and mechanisms. In addition, future studies should systematically determine the above-ground physiological indices of oilseed rape and soil oxygen content, and explore their intrinsic correlations with rhizosphere microbial communities and soil nitrogen dynamics. Moreover, future work can develop targeted clubroot control strategies (e.g., race-specific nitrogen fertilization, rhizosphere probiotic inoculants) based on these microecological findings to support practical oilseed rape production.

## Figures and Tables

**Table 1:** Effects of different physiological races on rhizosphere soil physicochemical properties.

Treatment	pH	TN (g/kg)	TP (g/kg)	TK (g/kg)	AN (mg/kg)	AK (mg/kg)	NO_3_^−^-N (mg/kg)	NH_4_^+^-N (mg/kg)	Ex-Ca (cmol/kg)	Ex-Mg (cmol/kg)
CK	7.05 ± 0.01 a	3.88 ± 0.01 c	0.62 ± 0.01 c	8.37 ± 0.01 c	421.78 ± 1.15 b	164.54 ± 0.01 b	26.24 ± 0.52 b	1.95 ± 0.27 c	1.35 ± 0.01 b	0.19 ± 0.00 b
CZ	6.65 ± 0.01 c	4.48 ± 0.01 b	0.70 ± 0.00 b	10.05 ± 0.07 a	496.09 ± 0.78 a	328.58 ± 0.87 a	28.88 ± 0.83 b	23.60 ± 0.51 a	1.34 ± 0.01 b	0.19 ± 0.00 b
KD	6.79 ± 0.01 b	4.56 ± 0.01 a	0.81 ± 0.01 a	8.98 ± 0.03 b	493.84 ± 1.01 a	325.76 ± 3.18 a	46.73 ± 0.14 a	6.64 ± 0.18 b	1.40 ± 0.00 a	0.21 ± 0.00 a

Note: The data in the table are presented as mean ± standard error (n = 3). Different letters in the same column indicate significant differences (*p* < 0.05). The same below.

**Table 2:** Richness and diversity of prokaryotic and eukaryotic microorganisms in rhizosphere soil.

Microbial	Treat	ACE Index	Chao1 Index	Shannon Index	Simpson Index	PD Whole Tree	Coverage
Bacteria	CK	2470.95 ± 116.44 c	2434.00 ± 121.63 c	9.47 ± 0.08 b	0.99 ± 0.00 a	164.53 ± 5.73 b	99.13
	CZ	2542.07 ± 110.52 b	2540.07 ± 150.29 b	9.34 ± 0.02 c	0.99 ± 0.00 a	164.65 ± 16.92 b	98.83
	KD	2725.88 ± 75.85 a	2724.74 ± 115.14 a	9.51 ± 0.06 a	0.99 ± 0.00 a	201.80 ± 9.03 a	98.67
Fungi	CK	400.09 ± 20.44 a	391.09 ± 20.90 a	4.72 ± 0.07 a	0.93 ± 0.00 a	90.34 ± 2.91 a	99.99
	CZ	217.28 ± 9.28 c	208.94 ± 9.97 c	2.73 ± 0.06 c	0.66 ± 0.01 c	46.40 ± 2.67 c	99.99
	KD	280.64 ± 10.85 b	281.38 ± 10.15 b	3.29 ± 0.08 b	0.71 ± 0.01 b	57.51 ± 2.54 b	99.99

Note: ACE and Chao1 indices reflect microbial species richness; Shannon and Simpson indices reflect microbial species diversity; PD whole tree reflects phylogenetic diversity; Coverage represents the sequencing coverage rate. Different letters in the same column indicate significant differences (*p* < 0.05).

## Data Availability

The original data presented in the study are openly available in Genome Sequence Archive in the National Genomics Data Center, China National Center for Bioinformation/Beijing Institute of Genomics, Chinese Academy of Sciences at https://ngdc.cncb.ac.cn/gsa, GSA: CRA037306 (public release on 19 January 2028).
